# Solid Indeterminate Nodules with a Radiological Stability Suggesting Benignity: A Texture Analysis of Computed Tomography Images Based on the Kurtosis and Skewness of the Nodule Volume Density Histogram

**DOI:** 10.1155/2019/4071762

**Published:** 2019-10-07

**Authors:** Bruno Max Borguezan, Agnaldo José Lopes, Eduardo Haruo Saito, Claudio Higa, Aristófanes Corrêa Silva, Rodolfo Acatauassú Nunes

**Affiliations:** ^1^Post-graduate Programme in Medical Sciences, School of Medical Sciences, State University of Rio de Janeiro, Rio de Janeiro, Brazil; ^2^Rehabilitation Sciences Post-graduate Programme, Augusto Motta University Centre (UNISUAM), Rio de Janeiro, Brazil; ^3^Division of Thoracic Surgery, Pedro Ernesto University Hospital, State University of Rio de Janeiro, Rio de Janeiro, Brazil; ^4^Technology Centre, Federal University of Maranhão, São Luis, Brazil; ^5^University Centre for Cancer Control, State University of Rio de Janeiro, Rio de Janeiro, RJ, Brazil

## Abstract

**Background:**

The number of incidental findings of pulmonary nodules using imaging methods to diagnose other thoracic or extrathoracic conditions has increased, suggesting the need for in-depth radiological image analyses to identify nodule type and avoid unnecessary invasive procedures.

**Objectives:**

The present study evaluated solid indeterminate nodules with a radiological stability suggesting benignity (SINRSBs) through a texture analysis of computed tomography (CT) images.

**Methods:**

A total of 100 chest CT scans were evaluated, including 50 cases of SINRSBs and 50 cases of malignant nodules. SINRSB CT scans were performed using the same noncontrast enhanced CT protocol and equipment; the malignant nodule data were acquired from several databases. The kurtosis (KUR) and skewness (SKW) values of these tests were determined for the whole volume of each nodule, and the histograms were classified into two basic patterns: peaks or plateaus.

**Results:**

The mean (MEN) KUR values of the SINRSBs and malignant nodules were 3.37 ± 3.88 and 5.88 ± 5.11, respectively. The receiver operating characteristic (ROC) curve showed that the sensitivity and specificity for distinguishing SINRSBs from malignant nodules were 65% and 66% for KUR values >6, respectively, with an area under the curve (AUC) of 0.709 (*p* < 0.0001). The MEN SKW values of the SINRSBs and malignant nodules were 1.73 ± 0.94 and 2.07 ± 1.01, respectively. The ROC curve showed that the sensitivity and specificity for distinguishing malignant nodules from SINRSBs were 65% and 66% for SKW values >3.1, respectively, with an AUC of 0.709 (*p* < 0.0001). An analysis of the peak and plateau histograms revealed sensitivity, specificity, and accuracy values of 84%, 74%, and 79%, respectively.

**Conclusions:**

KUR, SKW, and histogram shape can help to noninvasively diagnose SINRSBs but should not be used alone or without considering clinical data.

## 1. Introduction

Smoking is the most preventable cause of death globally, followed by cancer and cardiovascular disease. Many case series have considered lung cancer as the leading cause of cancer mortality [1]. Fortunately, increased access to computed tomography (CT) and newly recommended low-dose CT screening has facilitated the detection of new cases and helped reduce mortality [2].

Solitary pulmonary nodules (SPNs) are masses ≤3 cm surrounded by normal tissue. SPNs are generally asymptomatic and an incidental finding on the imaging results of the chest or upper abdomen [3, 4]. In fact, dozens of diseases might present in the nodular form, including infections such as tuberculosis, benign masses (e.g., hamartomas), and malignancies (e.g., primary or metastatic lung cancer). Unfortunately, a significant proportion of these nodules are classified as indeterminate, with an intermediate density between fat and calcium [3, 5]. For this type of nodule, the clinical stability criteria and risk factors for cancer are particularly important for diagnosis and treatment. According to the recommendations of the Fleischner Society, at least 2 years of tomographic follow-up study are required without significant changes to classify the nodule as benign [3–5].

International guidelines suggest that the diagnostic investigation of peripheral pulmonary nodules can be performed with both invasive transthoracic biopsy techniques (e.g., CT-guided transthoracic needle aspiration) and bronchoscopic techniques [6, 7]. Endoscopic techniques are useful for diagnosing peripheral (also benign) lesions, particularly when guided by newer navigational methods such as endoscopic ultrasound radial probes, electromagnetic navigation bronchoscopy, and others as well as when specific predictors of success are present (e.g., CT bronchus sign) [8]. Unfortunately, fine-needle aspiration biopsies of suspected benign nodules are relatively low in sensitivity and specificity because this method often cannot reach an aetiological diagnosis of the benign process; furthermore, it generates technical difficulties and complications, especially with regard to smaller nodules located deep in the lung parenchyma [9]. Hence, the need exists to use three-dimensional imaging technology to establish new lung nodule evaluation methods for more in-depth analyses of the textural features of the nodule by analysing the histogram data obtained on CT imaging [10].

With the onset of CT, nodule growth is often visualised more accurately through its largest diameter. Although a nodule diameter measurement seems sufficient and is commonly used, it cannot determine the spatial growth of the nodule across its several axes, which can cause confusion with regard to a nodule that maintains its diameter in the *X* and *Y* axes but progresses in the *Z* axis perpendicular to the other axes [11]. Some studies have shown that thin CT sections (approximately 1 mm) in the region of interest (ROI) of the nodule can determine its growth through volumetry [12, 13]. However, volume is not the only parameter measured because the texture can be evaluated with or without the administration of contrast [14].

In clinical practice, the evaluation of densities is frequently and briefly performed by delimiting a generally circular or elliptical area within an image (i.e., the ROI). One of the most simplified forms of texture analysis is the use of first-order grey-level statistics, which by definition uses the volumetric version of the pixel (voxel), one at a time. The three-dimensional structure of the voxel that results from the incorporation of thickness into the pixel provides information about radiological density, enabling its study [15, 16]. A recent study analysed 60 CT sets and demonstrated the textural heterogeneity of SPN, concluding that this calculation is useful when differentiating malignant from benign nodules [17]. In addition to the mean (MEN), other statistics of texture corresponding to the first-order grey levels rarely used in clinical practice such as kurtosis (KUR) and skewness (SKW) can be studied throughout the volume of the pulmonary nodules [18, 19]. Thus, the present study evaluated solid indeterminate nodules with a radiological stability suggesting benignity (SINRSBs) through a texture analysis of CT images.

## 2. Materials and Methods

A total of 50 CT scans of SINRSBs and 50 CT scans of malignant nodules from two image databases were retrospectively analysed. The research ethics committee at our institution approved the protocol under the number CAAE-36881414.1.0000.5259, and our procedure complies with current national and international standards.

### 2.1. CT-Scan-Acquisition Protocol

CT scans were obtained at our institution (University Hospital Pedro Ernesto of the State University of Rio de Janeiro, Rio de Janeiro, Brazil) using a helical acquisition apparatus (HiSpeed LX; General Electric Medical Systems, Milwaukee, WI, USA). The acquisitions were performed along the axial plane with the patients in the dorsal decubitus position using the following parameters: 120 kV, 100–200 mA (which varied according to the biotype of the patient) with automatic exposure control, a slice thickness of 1 mm, and a pitch of 2 mm from the jugular notch to the xiphoid process at full inspiration. The gantry was inclined by 43 cm. No intravenous contrast enhanced was administered during any of the examinations. After the scan acquisition, the CT images were reconstructed using a standard soft tissue kernel. In addition, a high-resolution reconstruction with a matrix of 512 × 512 points was performed using a high-frequency algorithm, a window width of 1,500 HU, and a MEN centre level of −700 HU.

### 2.2. Database

The SINRSB image database consisted of CT scans from 50 patients treated at our institution who presented with solid SPNs that met the stability criteria (minimal volume variation and volume doubling time >2,000 days) after at least 3 years of observation. Scans with the following characteristics were excluded: nodules with findings suggestive of benignity (total, central, lamellar, or popcorn calcification) and those with characteristics suggestive of malignancy (spiculation, lobulation, or high irregularity); nodules with total or partial ground-glass opacity; scans with more than one nodule; and scans that did not include sections with a thickness between 0.90 and 1.25 mm for the ROI of that nodule.

The image database of malignant nodules consisted of 50 CT scans whose diagnoses were confirmed via invasive methods and histopathology. These images were acquired from two databases, including that of our institution and that of the projects of the development of nodule detection programmes stored by the Cancer Imaging Archive (https://www.cancerimagingarchive.net), which is a server maintained by Siemens Healthcare™ that includes data from unidentified patients. The Cancer Imaging Archive is a platform seeks to provide data for the research and development of image processing methods. CT scans are available in downloadable DICOM format, and the portal provides spreadsheets that contain various image-acquisition information. To maintain radiomics robustness, we selected CT scans with a configuration similar to that of our institution's database (including CT mode, no contrast enhancement, a section thickness between 0.9 and 1.25 mm, a matrix of 512 × 512 points, and a soft tissue reconstruction kernel).

### 2.3. Segmentation and Imaging Processing

The images were quantised in 12 bits (i.e., the equivalent of 12 grey levels) [15] and were later stored in DICOM format. The digital data were transferred to Bebúi software (Technological Centre, Federal University of Maranhão, São Luis, MA, Brazil) where the extraction of radiomic features based on the ROI was completed using in-house texture analysis algorithms. The images were evaluated using Bebúi software by fellowship-trained readers (B.M.B. and R.A.N. with 14 and 20 years of experience, respectively). An ROI was carefully drawn in the nodule avoiding contact with its edges using a semiautomated process. The software provides tools that digitally subtract vessels, bronchi, fibrosis scars, and other structures that do not belong to the nodule for all of the slices that correspond to its volume. The software also shows the region growing processing and segmentation algorithm installed through a “seed” to generate a database of that nodule with volume and histogram data. During this process, if motion, contrast streaking, or beam-hardening artefacts were noted on the image with maximal dimensions, then another “artefact-free” image demonstrating the lesion was chosen.

Using the software's dialog box, the interval was defined in Hounsfield units (HUs) between −450 and +1500. The seed voxel was placed within the nodule that analysed the neighbouring voxels using a 3D region growing algorithm [17] for inclusion based on the contrast intervals and number of slices determined in the dialog box. The location of the seed in the slice was automatically revealed based on the two-dimensional coordinates *X* and *Y*.

After SPN segmentation, the radiomics features were extracted for each nodule, including first-order statistics to assess the distribution of CT or voxel values. Using the CT scans, KUR and SKW were determined for the whole volume of each nodule. By definition, KUR is the property of a frequency distribution that characterises its flattening relative to the Gaussian curve (i.e., it determines the degree of flattening of a distribution curve) [18]. Thus, KUR measures the peakedness of the distribution of values and is considered as a marker of vascularity and tumour angiogenesis, which in turn are essential factors that determine tumour aggressiveness and overall survival [19]. High KUR is related to several outliers, whereas low KUR suggests a lack of outliers [18, 19]. A given curve is symmetrical when the distribution of values around the centre point is exact (i.e., the MEN, median, and mode coincide). The deviation from symmetry is measured as SKW, which indicates the asymmetry in the distribution of voxel intensities [18].

KUR is mathematically defined as the fourth moment of statistical distributions and, unlike the as the standard deviation, should not be considered as a measure of dispersion; rather, it is a distribution model. KUR is as a mass movement that does not affect the variance (VAR). Positive KUR is characterised as the presence of a peak with heavy tails, whereas negative KUR is characterised by lighter tails and a flatter peak than the normal distribution [20].

KUR and SKW were calculated using the following formulas:

(1) $$\begin{array}{c} \text{KUR}=\frac{1}{{\text{VAR}}^{4}}\overset{G-1}{\underset{i=0}{\sum }}{\left(i-\text{MEN}\right)}^{4}\text{HU}-3, \end{array}$$

(2) $$\begin{array}{c} \text{SKW}=\frac{1}{{\text{VAR}}^{3}}{\left\{\overset{G-1}{\underset{i=0}{\sum }}{\left(i-\text{MEN}\right)}^{3}\text{HU}\right\}}^{2}, \end{array}$$

where KUR = kurtosis, SKW = skewness, VAR = variance, MEN = mean, and HU = Hounsfield unit.

Additionally, CT scans were used to evaluate the histogram and obtaining data on the SPN texture patterns. Thus, Portable Network Graphics (PNG) files were generated using Python's Matplotlib tool (https://www.matplotlib.org).

Using Bebúi software, the inter-observer reliabilities of the images acquired were compared with 25 retrospectively segmented images for extraction of ROI-based morphological features by two independent readers (B.M.B. and R.A.N.) in a blinded form. These readers segmented the images and processed the histogram data for KUR and SKW without a front end. The semiautomatic calculation process minimized human intervention during the various histogram data processing events.

### 2.4. Statistical Analyses

Statistical analyses were performed using SPSS 16 (Chicago, IL, USA), which automatically generated the receiver operating characteristic (ROC) curve and calculated the best cut-off point by establishing test and state variables. The histograms were analysed and separated into two patterns: (1) the peak pattern (i.e., leptokurtic) in which the data are symmetrical or skewed but with a predominance of an increase in values; and (2) the plateau pattern (i.e., platykurtic) in which the MEN was distributed within regular intervals without a sudden increase of the data. Interclass correlation coefficients (ICCs) were employed to study the reliability of radiomic features, and an ICC was considered as acceptable if it was ≥0.85 [21]. Statistical significance was considered as *p* < 0.05.

## 3. Results

The interobserver reliabilities for KUR and SKW were high (ICCs of 0.87 ± 0.10 and 0.91 ± 0.12, respectively). Regarding the data extracted for analysis, however, we used the data agreed upon by both readers.

**Figure 1:**
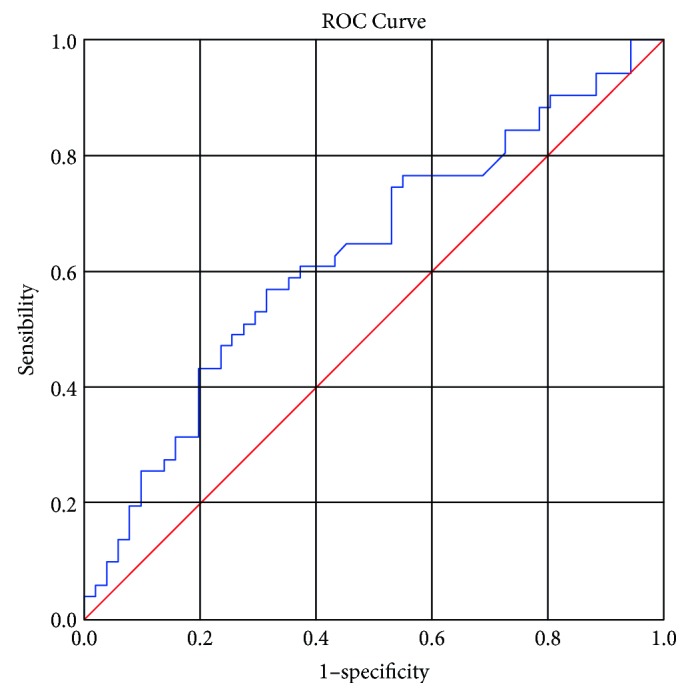
ROC curve for KUR, AUC = 0.709 (*p* < 0.0001). The diagonal segments were produced by ties.

The MEN KUR of the 50 SINRSBs and malignant nodules were 3.37 ± 3.88 and 5.88 ± 5.11, respectively. The ROC curve was obtained, showing that when KUR >6, the sensitivity and specificity for distinguishing between malignant nodules and SINRSBs were 65% and 66%, respectively, with an area under the curve (AUC) of 0.709 and *p* < 0.0001 (Figure 1).

**Figure 2:**
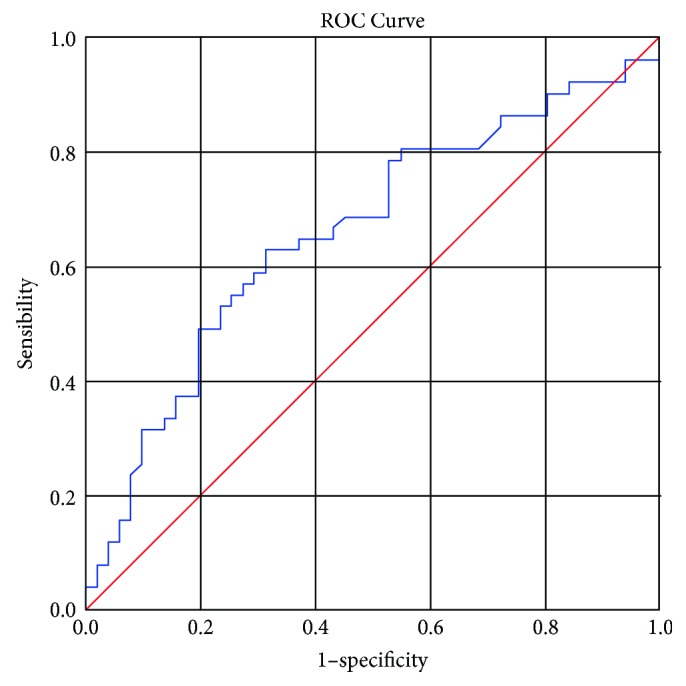
ROC curve for SKW, AUC = 0.705 (*p* < 0.0001). The diagonal segments were produced by ties.

The MEN SKW of the 50 SINRSBs and malignant nodules were 1.73 ± 0.94 and 2.07 ± 1.01, respectively. The ROC curve enabled the establishment of a cut-off point that best fit the sensitivity and specificity values of the SKW, where 3.1 was the cut-off point for malignant nodules and SINRSBs. This value was associated with a sensitivity of 62.7%, a specificity of 69%, and an AUC of 0.705 (*p* < 0.005) to differentiate malignant nodules from SINRSBs (Figure 2).

**Figure 3:**
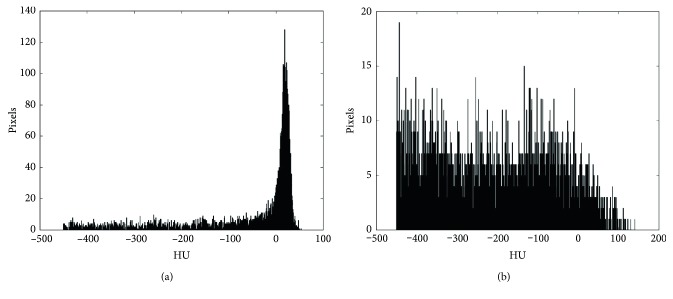
Histogram of a malignant nodule with a leptokurtic (i.e., resembling a peak) and negatively skewed distribution (a); histogram of a solid indeterminate nodule with a radiological stability suggesting benignity with a platykurtic distribution curve (i.e., resembling a plateau) and lower SKW (b).

Additionally, a histogram analysis was performed between the peak and plateau patterns. Typical aspects of SINRSBs and malignant nodules are shown in Figure 3. Among the 50 malignant nodules, 42 presented with the peak pattern, and only eight presented with the plateau pattern. Among the 50 SINRSBs, however, 37 presented with the plateau pattern, and only 13 showed the peak pattern. The histogram analysis between the peak and plateau patterns revealed a sensitivity of 84%, a specificity of 74%, and an accuracy of 79%.

## 4. Discussion

The present study revealed that a texture analysis of the CT images of patients with SINRSBs using easy-to-use software aids in the assessment the nature of the lesion. The KUR, SKW, and graphical analysis of histogram patterns might help differentiate SINRSBs from malignant nodules in these patients.

Radiomics (the study of extracting computerised, algorithm-based features to quantify the phenotypic characteristics of lesions using medical images) [22, 23] has been used to construct predictive models that relate image characteristics to tumour characteristics. Its four quantitative descriptive characteristics are morphological, statistical, regional, and model-based [23]. Several recent studies have used KUR and SKW as diagnostic indicators for various purposes, including to analyse liver fibrosis due to hepatitis C virus infection [24], central nervous system injuries with tumour differentiation [25], substantia nigra lesions due to carbon monoxide exposure [26], pancreatic tumour type differentiation [27, 28], and the association between metabolic patterns and several types of lesions in patients with cervical carcinoma [29]. Son et al. [30] collected tomographic measurements of lung tissue that included the KUR and SKW values associated with ground-glass nodules with or without solid components. These authors found that statistical analyses are a useful tool for differentiating invasive adenocarcinoma from pre-invasive and minimally invasive adenocarcinoma. More recently, Yagi et al. [31] performed a similar study that confirmed the potential of this method for differentiating minimally invasive adenocarcinoma from invasive adenocarcinoma [31].

Given the high morbidity and mortality associated with lung cancer, differentiating benign nodules from malignant nodules is crucial [22]. Alpert et al. [32] evaluated nodules with a lepidic pattern. In that study, 3D volumetry and first-order grey-level statistics obtained a sensitivity of 81% and a specificity of 76.7% when differentiating lepidic lesions from invasive lung adenocarcinoma. In another study, Kamiya et al. [33] examined the KUR and SKW of solid nodules to differentiate malignant from benign nodules. Similar to our results, those authors noted that KUR tends to be higher among malignant nodules than benign nodules. The AUC values of the ROC curve ranged from 0.71 to 0.83, and these results were similar to those of our study.

The CT pixels or voxels that comprise the image are the result of X-ray beam attenuation as it passes through a small portion of living tissue [2, 10]. The behaviours of the various attenuations are used to construct the histogram, and its study is only another step in the process of differentiating SINRSBs from malignant nodules, without ignoring the initial appearance (spicules, lobules, MEN diameter, and visceral pleural retraction) that, when combined with the radiologist's experience and the support of clinical data, contributes to the definition of probable malignancy. If doubt persists and the nodule is classified as indeterminate, then volumetric monitoring and the determination of the volume-doubling time are recommended [2–4]. In this context, the histogram that does not use a contrast can be useful, especially for patients who present with characteristics that prevent the administration of radiological contrast and hinder follow-up assessment. Interestingly, the histogram plots presented in our study demonstrate that malignant nodules showed a spiculated pattern ranging from 0 to 100 HU, which corresponds to the range of predominant protein tissues such as soft tissue.

The present study found that KUR and SKW can be used to differentiate SINRSBs from malignant benign nodules. Because the sensitivity and specificity values were close to 65%, however, KUR and SKW should not be used alone; rather, they should be combined with other parameters. Therefore, volumetric assessment and volume doubling time remain key elements in the evaluation of indeterminate pulmonary nodules [34, 35]. More recently, Mao et al. [23] evaluated the usefulness of a radiomic predictive model developed from baseline low-dose CT screening. These authors observed that the sensitivity and specificity for predicting malignancy in SPNs were 81% and 92.2%, respectively. In that study, the benign nodules had greater SKW and less KUR compared with malignant nodules.

For efficient data processing, it was necessary to develop a specific tool that, in addition to aiding the calculation of KUR and SKW, generated PNG files using the Matplotlib tool for Python. Thus, it was possible to separate peak and plateau patterns. In addition to the calculating KUR and SKW, the graphical appearance of the histograms for these two patterns helped differentiate nodules into groups of SINRSBs and malignant nodules with an accuracy of 79%. The sensitivity and specificity values were encouraging in terms of the viable contribution of this method for identifying malignant lesions. However, it remains possible that the high protein level of the malignant nodules is related to increased protein synthesis by the cells (i.e., the small tissue portion that corresponds to a voxel in the image might also correspond to a group of cells with high protein synthesis). In this respect, positron emission tomography (PET) has demonstrated greater metabolic activity with protein synthesis in malignant lesions.

Obviously, benign nodules can exhibit high-density tissue masses (e.g., fat) and high-volume calcifications (e.g., hamartomas), thereby generating HU peaks. However, because the nodules in the present study were indeterminate and stable, their composition was likely more homogeneous and with less rich material in the cells. Naturally, this finding does not suggest that these nodules do not experience slower changes over longer periods [36]. Interestingly, recent studies have shown that tumour heterogeneity estimation using the distribution of pixel values with radiomic features can be used as a marker of tumour aggressiveness and treatment response in this patient population [37, 38]. In particular, KUR might be useful for predicting and assessing response to antiangiogenic treatment among patients with lung cancer [19]. More recently, Digumarthy et al. [19] demonstrated that a radiomics evaluation adds incremental value to one's clinical history and standard imaging features in predicting histology (i.e., distinguishing squamous and adenocarcinoma subtypes of nonsmall cell lung cancers) and epidermal growth factor receptor mutations.

One strength of our study is that we believe it is the first to show how histogram patterns differ between SINRSBs and malignant pulmonary nodules, likely contributing to the tomographic study of these lesions. As with any other study, however, ours also has limitations. First, the sample size was relatively small, although the size was justified by the strict imaging criteria used to exclude characteristically benign and malignant lesions to only evaluate indeterminate pulmonary nodules. Second, the benign nodules had no histological diagnoses, and these diagnoses were made only at the radiological level based on stability criteria; thus, we chose to call these cases “SINRSB”. Third, the radiomics reproducibility likely depends on the amount of data and the consistency of the parameters used to produce the images; therefore, slight changes in the imaging dataset can greatly affect the robustness of the radiomic features [23, 39]. In our study, the SINRSB data comprised a single image database, whereas the malignant nodule dataset included several image databases obtained outside a follow-up setting. Although the chest CT protocol might affect the radiomics results, importantly, no recommendations exist for a standardised protocol to evaluate SPN radiomics, which limits our ability to compare studies and affects the generalisation of radiomic analysis [39]. Despite these limitations, we believe that our results justify additional research on the use of CT image texture analysis, especially with regard to peak and plateau patterns.

In conclusion, the present study shows that KUR, SKW, and the general appearance of the histogram contribute to the noninvasive diagnoses of SINRSBs and malignant nodules. However, these characteristics should not be used alone or without considering clinical data.

## Data Availability

The data used to support the current findings are available from the corresponding author upon request.

## Abbreviations

AUC: Area under the curve
ICC: Interclass correlation coefficient
CT: Computed tomography
DICOM: Digital imaging communications in medicine
HU: Hounsfield units
KUR: Kurtosis
MEN: Mean
PET: Positron emission tomography
PNG: Portable network graphics
ROC: Receiver operating characteristic
ROI: Region of interest
SINRSB: Solid indeterminate nodules with a radiological stability suggesting benignity
SKW: Skewness
SPN: Solitary pulmonary nodule
VAR: Variance.
